# Association between dietary fiber intake and obesity in US adults: from NHANES 1999–2018

**DOI:** 10.3389/fnut.2025.1602600

**Published:** 2025-07-07

**Authors:** Siran Lai, Yuning Zeng, Geer Lin, Yue Li, Zixian Lin, Xueren Ouyang

**Affiliations:** ^1^Department of Pediatrics, Shunde Women and Children's Hospital of Guangdong Medical University, Foshan, China; ^2^Institute of Traditional Chinese Medicine,Shunde Women and Children's Hospital of Guangdong Medical University, Foshan, China; ^3^Guangzhou University of Chinese Medicine, Guangzhou, China

**Keywords:** dietary fiber intake, obesity risk, mortality, NHANES dataset analysis, nutrition

## Abstract

**Background:**

Previous studies have demonstrated that dietary fiber reduces the incidence of diabetes or hyperlipidemia, but it remains unclear how dietary fiber intake relates to obesity incidence.

**Methods:**

A total of 39,184 adults were obtained from the National Health and Nutrition Examination Survey (NHANES) from 1999 to 2018. Multifactorial logistic regression analysis and Cox regression analysis were used to investigate the correlation between dietary fiber intake and obesity. Restricted cubic spline (RCS) analyses were used to describe the dose–response correlation between dietary fiber intake and the incidence of obesity. The robustness of the results was enhanced by Kaplan–Meier survival analyses and subgroup analyses.

**Results:**

After adjusting for confounders, dietary fiber intake in the quartile 4 (≥ 20.8 g/day) was linked with a 26% lower incidence of obesity than those in the quartile 1 [≤9.1 g/day; odds ratios (OR) = 0.74, 95% confidence interval (95% CI): 0.67–0.83, *p* < 0.0001]. Further study indicated that a heightened dietary fiber intake was linked with a 21% decrease in all-cause mortality in quartile 4 compared to quartile 1 [hazard ratio (HR) = 0.79, 95% CI: 0.65–0.96, *p* = 0.02]. The RCS analysis conspicuously showed a non-linear U-shaped association between dietary fiber intake and all-cause mortality (*p* < 0.05), with 26.3 g/day being the turning point. The HR curve for all-cause mortality initially decreased and then increased.

**Conclusion:**

Adequate dietary fiber intake has a favorable effect on reducing the incidence of obesity events, and obese patients with high dietary fiber intake exhibit lower all-cause mortality.

## 1 Introduction

Obesity is a global health problem that affects individuals of all ages and is linked to the emergence of various chronic diseases (1), for example, cancer (2), cardiovascular disease (3), and diabetes (4). With lifestyle changes, the incidence of obesity continues to rise globally (5). According to data estimated by the World Health Organization in 2022, more than 2.5 billion adults were overweight worldwide, of whom approximately 890 million were obese (6). This underscores the severity of obesity as a major public health challenge.

Nutritional intervention is crucial in obesity treatment. Multiple dietary patterns, such as the Mediterranean diet (7), low-carbohydrate diet (8), and vegetarian diet (9), have all been proven to be effective for weight loss. The prospective cohort study by Liping Lu et al. indicated that calcium, an essential nutrient for the human body, is inversely associated with the incidence rate of obesity (10). Furthermore, it is worth mentioning that the systematic review by Cadeyrn J. Gaskin et al. analyzed 38 weight management guidelines worldwide, and all of them unanimously endorsed nutritional interventions and physical activity as foundational components of obesity treatment (11). Dietary fiber is an essential nutrient for human health and a key component in nutritional interventions (12), playing a significant role in maintaining intestinal health, regulating blood glucose and lipid levels, and promoting intestinal motility (13). Nevertheless, there is a significant discrepancy between the actual dietary fiber intake and the recommended standards. The United States Department of Agriculture’s Dietary Guidelines for Americans 2020–2025 recommend a daily dietary fiber intake that varies by age and gender. For individuals under 51 years of age, the suggested daily intake is 25 g for women and 38 g for men. For those 51 years and older, women should aim for 21 g per day, and men should target 30 g. However, approximately 97% of men and 90% of women do not have the suggested intake of dietary fiber (14). This widespread insufficiency in intake is associated with elevated risks of multiple diseases, while increased dietary fiber consumption has been demonstrated to effectively prevent such conditions. These include obesity-related complications such as diabetes (15), hypertension (16), metabolic syndrome (17), coronary heart disease (18), hyperlipidemia (19), and so on. Furthermore, Fatemeh et al. found that higher dietary fiber intake was linked with a decreased likelihood of mortality from all causes and mortality due to cardiovascular disease and cancer (20). Although the benefits of adequate dietary fiber intake for most diseases have been well-established, the relationship between dietary fiber intake and obesity has not yet been fully elucidated. Current research has primarily focused on metabolic indices or short-term interventions, with a deficiency in long-term obesity incidence data derived from large-scale populations. Moreover, whether mortality conclusions from the general population can be extrapolated to obese individuals requires further validation.

As a result, this study utilizes the National Health and Nutrition Examination Survey (NHANES) database (1999–2018) to examine the associations between dietary fiber intake and obesity, providing a robust evidence base for preventive nutritional strategies against obesity and clinical decision-making in obesity management. We hypothesize that dietary fiber intake is inversely associated with the incidence of obesity and that a high intake of dietary fiber can reduce the all-cause mortality risk in obese individuals.

## 2 Materials and methods

### 2.1 Study design and population

The data used in our study were sourced from the NHANES, a comprehensive dataset on health and nutrition managed by the National Center for Health Statistics. Starting in 1999, NHANES has been conducting continuous surveys, collecting annually representative samples of health and nutrition status data from approximately 5,000 individuals across the United States. Each cycle’s dataset encompasses various types of information, such as questionnaire data on demographics, socio-economic status, diet, and health issues, as well as physical examination components, such as physiological measurements and laboratory tests. The study got approval from the National Center for Health Statistics Research Ethics Review Committee. The NHANES project protocol strictly adheres to the ethical principles outlined in the Declaration of Helsinki. The cross-sectional study employed in this research strictly follows the Strengthening the Reporting of Observational Studies in Epidemiology reporting guidelines (21).

**Figure 1:**
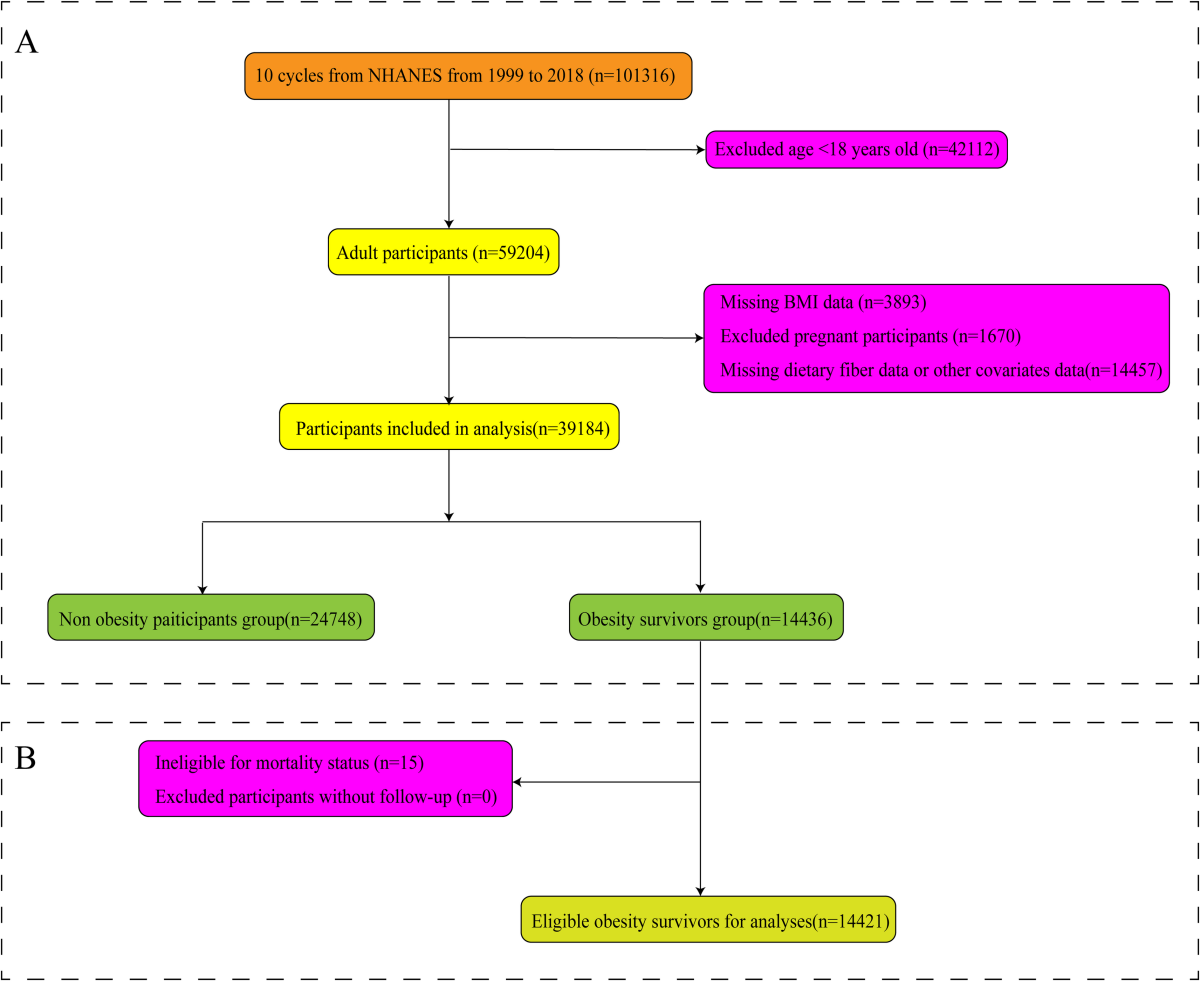
Flowchart of participants’ selection. Association of fiber intake with obesity **(A)** and mortality **(B)** among patients with obesity.

In our study, the data employed were from 10 survey cycles spanning from 1999 to 2018. Part I: We evaluated 101,316 participants from 10 consecutive cycles of the NHANES. Initially, we excluded data from participants younger than 18 years of age (*n* = 42,112) and those who were pregnant (*n* = 1,670). Subsequently, we excluded data lacking body mass index (BMI) information (*n* = 3,893). Finally, we excluded missing values for dietary fiber and covariates, including ethnicity, age, gender, energy intake, education level, poverty income ratio (PIR), marital status, carbohydrate intake, diabetes status, alcohol consumption, hypertension status, occupational physical activity, smoking status, and recreational physical activity (*n* = 14,457). Ultimately, 39,184 participants were included to study the cross-sectional correlation between dietary fiber intake and obesity. Among them, 14,436 individuals had a BMI of 30 kg/m^2^ or greater, meeting the diagnostic criteria for obesity (Figure 1A). Part II: To further investigate the longitudinal relationship between mortality and dietary fiber intake among obese patients, in our analysis, we excluded 15 participants due to a lack of survival data. Ultimately, 14,421 eligible adults were included in our survival analysis assessment (Figure 1B). In our analyses, the median follow-up duration for adult participants was 9.08 years, providing a sufficiently long period for assessing outcomes. All participants submitted written informed consent before taking part in the study. In this study, the NHANES dataset used is publicly available; thus, no additional ethical or administrative approval was required.

### 2.2 Assessment of obesity

BMI (calculated by dividing weight in kilograms by the square of height in meters) was used to define obesity as an indicator (22). In the Mobile Examination Center (MEC), health professionals, trained using calibrated equipment and standardized protocols, measured weight and height. Based on the classification by the Centers for Disease Control and Prevention (23), adults were categorized as underweight (BMI < 18.5), normal weight (BMI 18.5 to <25), overweight (BMI 25.0 to <30), or obese (BMI ≥ 30) (24). It should be noted that participants with a BMI < 30 were categorized as non-obese in this study.

### 2.3 Assessment of dietary fiber intake

The primary variable was the intake of dietary fiber. Dietary recall interviews conducted from 1999 to 2020 were conducted in person by trained dietary interviewers who were fluent in both Spanish and English. The setting of the interview is a private room in the MEC. Each MEC dietary interview room contains a standard set of measuring guides. Participants were provided with measuring cups, spoons, rulers, and food model booklets containing two-dimensional illustrations to report food quantities during recall interviews. To obtain a more complete picture of the usual dietary intake of the US population, a second dietary interview for all participants who complete the first recall was added to the survey in 2002. The “What We Eat in America” data were collected using the USDA’s dietary data collection instrument, the Automated Multiple-Pass Method, available at: http://www.ars.usda.gov/nea/bhnrc/fsrg. The Automated Multiple-Pass Method was designed to provide an efficient and accurate means of collecting dietary intakes for large-scale national surveys. The Automated Multiple-Pass Method is a fully computerized recall method that uses a five-step interview process outlined below: 1. Quick List: Participants recall all foods and beverages consumed the day before the interview (from midnight to midnight). 2. Forgotten Foods: Participants are asked about the consumption of foods commonly forgotten during the Quick List. Step. 3. Time and Occasion: The time and eating occasion are collected for each food. 4. Detail Cycle: For each food, a detailed description, amount eaten, and additions to the food are collected. Eating occasions and times between eating occasions are reviewed to elicit forgotten foods. 5. Final Probe: Additional foods not remembered earlier are collected. Dietary fiber intake was determined by NHANES, which employs a rigorous data collection protocol. This includes the use of food composition tables to quantify nutrient intake. The NHANES Dietary Interview Procedures Manual^1^ shows a complete and detailed methodology. Similar to previous studies (25), we categorized the percentage of dietary fiber intake into quartiles and selected the dietary fiber (g) on the first day, which was collected from the 24-h first-meal recall interview conducted face-to-face at the MEC and adjusted for individual weight factors. The sources of dietary fiber, including vegetables, cereals, and fruits, are determined by classifying the relevant food categories (26).

### 2.4 Mortality outcomes

The principal outcome of this study is all-cause mortality. Up to 31 December 2019, NHANES supplied mortality data connected to the National Death Index. We investigated both mortality of all-cause and specific causes, comprising malignant neoplasms (ICD-10: C00-C97) and heart disease (ICD-10: I00-I09, I11, I13, I20-I51). The baseline data collection of NHANES marks the beginning of calculating survival time. The time for participants who did not experience the event censored at 31 December 2019, or who had elapsed time from the interview date to the date of death, was deemed as follow-up time.

### 2.5 Covariate assessment

We comprehensively screened 13 potential confounding factors associated with obesity, namely age, ethnicity, gender, PIR, work activities, education level, marital status, energy intake, hypertension status, recreational activities, smoking status, alcohol consumption, and diabetes status, which were determined as risk factors. The confounding factors included in our analysis were carefully chosen according to their established correlation with obesity incidence and their common inclusion in diet and obesity studies conducted by NHANES (27). This selection ensured that our results were adjusted for variables that could potentially confound the correlation between obesity incidence and dietary fiber intake. Gender was male and female. The patients’ ages ranged from 20 to 85 years (28). Ethnicity was Mexican American and non-Hispanic White, non-Hispanic Black people, other Hispanic, and other ethnicities (29). Education level was classified as less than high school, high school, and more than high school (30). The PIR was classified as ≤1.0, 1.0–3.0, and >3.0 (31). Marital status was divided into married/living with a partner, widowed/divorced/separated, and never married by the latest NHANES classification (32). Common risk factors included hypertension, diabetes (33), smoking history (34), and alcohol consumption history (classified as yes/no) (35). Hypertension and diabetes were diagnosed by specific criteria, including medical diagnosis, biochemical indicators, and medication use. Weight, height, and alcohol consumption were recorded at the MEC. Individuals who consumed at least 12 times per year were characterized as drinkers. In their lifetime, individuals smoked less than 100 cigarettes or never smoked were characterized as never smokers. Physical activity was mainly classified into work activities and recreational activities, and we classified work activities and recreational activities as yes/no based on questionnaire responses.

### 2.6 Statistical analysis

All analyses in our study were conducted using R version 4.2.0. We utilized the complex sampling weights (Day 1 dietary sample weights) recommended by the Centers for Disease Control and Prevention. We integrated sample weights from 10 consecutive cycles, following the methods suggested on the NHANES website. Continuous variables in the baseline characteristics table are displayed as survey-weighted means with standard errors (SE), and classified variables are shown as sample sizes with survey-weighted percentages.

In our analysis, logistic regression was employed to ascertain the relationship between dietary fiber intake and obesity incidence, which is a generalized linear model with a logit link function. The initial model was designed as a univariate analysis without adjusting for any covariates, providing a baseline assessment of the correlation between obesity incidence and dietary fiber intake. Model 1 introduced demographic variables, controlling for ethnicity, gender, and age, to account for basic population characteristics. Building upon this, Model 2 was additionally adjusted for both socioeconomic and lifestyle factors, including marital status, PIR, and education level, which are known to affect health outcomes. Finally, Model 3 comprehensively adjusted for all identified confounding factors, such as energy intake, diabetes status, hypertension status, smoking status, alcohol consumption, work activities, and recreational activities, to ensure a reliable assessment of the correlation between dietary fiber intake and obesity incidence. Restricted cubic spline (RCS) curves investigated the dose–response correlation between obesity incidence and dietary fiber intake. Additionally, we performed survey-weighted multivariate logistic regression and subgroup analyses, categorizing dietary fiber intake into four groups based on intake percentages. Subgroup analyses were pre-specified based on theoretical considerations and prior literature. These analyses were stratified by gender, age, PIR, marital status, ethnicity, work activities, education level, diabetes status, recreational activities, smoking status, alcohol consumption, and hypertension status to estimate the uniformity of the correlation between obesity incidence and dietary fiber intake across different subgroups.

Kaplan–Meier survival analysis and Cox proportional hazards models were employed to evaluate the correlation between dietary fiber intake and all-cause mortality, mortality of cardiac mortality, and cancer mortality. The dose-response relationship between mortality risk and dietary fiber intake was estimated and visualized using RCS models, with separate RCS analyses conducted for all-cause, cardiac, and cancer mortality. The subgroup analyses we conducted further assessed the relationship between dietary fiber and mortality across different subgroups. The analyses included stratification by gender, age, PIR, ethnicity, marital status, education level, hypertension status, work activities, diabetes status, recreational activities, smoking status, and alcohol consumption. Multiplicative interaction terms between subgroups were incorporated into the models to evaluate potential interaction effects. When the *p*-value was less than the threshold of 0.05, statistical analyses were considered statistically significant in this study.

## 3 Result

### 3.1 Characteristics of participants

In total, 101,316 participants were enrolled in this study from 10 cycles (1999–2018) of the NHANES database. After screening, 39,184 participants were ultimately included in the data analysis. A weighted baseline table for the population was created based on whether participants had a history of obesity, dividing them into non-obese group (*n* = 37,555) and obese group (*n* = 1,455; Table 1). The mean age (SE) of all participants was 47.15 (0.21) years, and the mean (SE) dietary fiber intake was 16.64 (0.12) g/day. Among them, a relatively larger proportion (50.82%) were female participants, 70.51% were non-Hispanic White, 69.67% had a high level of education, 35.58% had a PIR level greater than 3.0, 62.91% were married/cohabiting, 46.84% were smokers, 74.66% consumed alcohol, 12.75% had diabetes, and 37.78% had hypertension. We also summarized the basic characteristics of the obese population, which mostly consisted of older individuals, female participants, individuals with a higher level of education, individuals with moderate income, smokers, alcohol consumers, and those with hypertension. The mean age (SE) of the obese population was 48.15 (0.23) years, and the mean (SE) dietary fiber intake was 15.80 (0.14) g/day, which was lower than that of the non-obese population (Supplementary Table S1). The weighted overall prevalence of obesity was 36.84%, and its basic characteristics are presented in Table 1.

**Table 1:** Characteristics of the study population sorted by obesity.

Characteristic	Overall	Non-obesity	Obesity	*p*-value
N	39,184	24,748	14,436	
Age, years	47.15(0.21)	46.60(0.25)	48.15(0.23)	< 0.0001
BMI(kg/m^2^)	28.80(0.07)	24.86(0.03)	35.86(0.08)	< 0.0001
Sex, n (%)				< 0.001
Female	19,480(50.82)	11,581(49.78)	7,899(52.69)	
Male	19,704(49.18)	13,167(50.22)	6,537(47.31)	
Ethnicity, n (%)				< 0.0001
Non-Hispanic White	18,376(70.51)	12,071(71.91)	6,305(68.02)	
Non-Hispanic Black people	8,022(10.54)	4,356(8.85)	3,666(13.58)	
Mexican American	6,575(7.68)	3,936(6.99)	2,639(8.90)	
Other ethnicities	6,211(11.27)	4,385(12.26)	1826(9.50)	
Education level, n (%)				0.04
Less than high school	4,233(5.08)	2,695(5.13)	1,538(5.00)	
High school	11,277(25.25)	7,020(24.68)	4,257(26.27)	
More than high school	23,674(69.67)	15,033(70.20)	8,641(68.74)	
PIR, n (%)				< 0.0001
≤1.0	7,743(13.91)	4,771(13.49)	2,972(14.66)	
1.0–3.0	15,001(50.51)	9,845(52.18)	5,156(47.50)	
>3.0	16,440(35.58)	10,132(34.32)	6,308(37.84)	
Marriage, n (%)				< 0.0001
Divorced/separated/widowed	8,729(18.88)	5,293(18.10)	3,436(20.27)	
Married/living with partner	23,667(62.91)	14,960(62.69)	8,707(63.32)	
Never married	6,788(18.21)	4,495(19.21)	2,293(16.41)	
Drinking status, n (%)				< 0.0001
No	12,289(25.34)	7,280(23.36)	5,009(28.89)	
Yes	26,895(74.66)	17,468(76.64)	9,427(71.11)	
Smoking status, n (%)				0.4
No	20,872(53.16)	13,023(52.92)	7,849(53.60)	
Yes	18,312(46.84)	11,725(47.08)	6,587(46.40)	
Diabetes mellitus, n (%)				< 0.0001
No	32,444(87.25)	21,813(92.25)	10,631(78.28)	
Yes	6,740(12.75)	2,935(7.75)	3,805(21.72)	
Hypertension, n (%)				< 0.0001
No	22,424(62.22)	15,824(69.34)	6,600(49.47)	
Yes	16,760(37.78)	8,924(30.66)	7,836(50.53)	
Work activity, n (%)				0.25
No	20,210(45.44)	12,782(45.07)	7,428(46.09)	
Yes	18,974(54.56)	11,966(54.93)	7,008(53.91)	
Recreational activity, n (%)				< 0.0001
No	23,384(54.33)	14,042(50.46)	9,342(61.25)	
Yes	15,800(45.67)	10,706(49.54)	5,094(38.75)	
Energy intake (kcals/day)	2174.48(7.45)	2190.19(9.13)	2146.33(11.83)	0.003
Fiber intake (g/day)	16.64(0.12)	17.11(0.15)	15.81(0.14)	< 0.0001

Continuous variables are shown as weighted means ± SE. Categorical variables are shown as unweighted counts (weighted percentages).

### 3.2 Association between dietary fiber intake and obesity incidence

**Figure 2:**
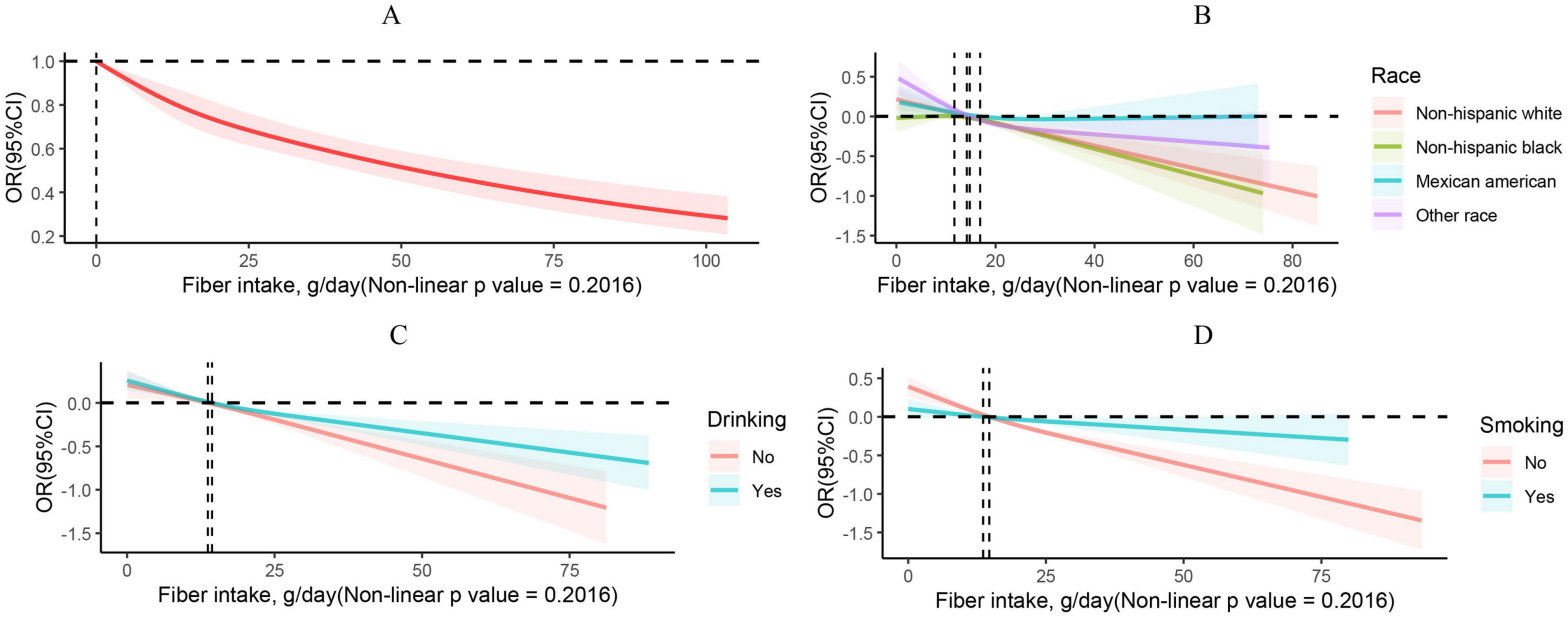
RCS curves describing the dose–response relationship between fiber intake and obesity **(A)**. RCS curves describing the dose–response relationship between fiber intake and obesity by grouping based on ethnicity **(B)**, drinking **(C)**, and smoking **(D)**.

Using multifactorial logistic regression analysis, the correlation between dietary fiber intake and incidence of obesity was evaluated. A negative correlation between dietary fiber and obesity was found, which may reduce the risk of obesity (Table 2). As a continuous variable, in fully adjusted models, dietary fiber intake was negatively correlated with obesity (odds ratios (OR) = 0.99; 95% confidence interval (95% CI), 0.98–0.99, *p* < 0.0001). This negative correlation was also observed in other models. As a categorical variable, based on quartiles of dietary fiber percentage, all of the models demonstrated that dietary fiber intake had a protective effect against obesity. In the fully adjusted model, participants in quartiles 2, 3, and 4 had a reduced risk of obesity by 9, 18, and 26%, respectively, compared to those in quartile 1 (OR = 0.91; 95% CI, 0.84–0.99, *p* = 0.03), (OR = 0.82; 95% CI, 0.75–0.89, *p* < 0.0001), and (OR = 0.74; 95% CI, 0.67–0.83, *p* < 0.0001). The same conclusion was observed in the original model, Model 1, and Model 2. The trend *p*-values for all the above models were less than 0.001, indicating that as dietary fiber intake increases, the incidence of obesity tends to decrease, providing a protective effect. The strongest protective effect was most prominently observed in quartile 4 among all models, which corresponds to a dietary fiber intake greater than 20.8 g/day, with the lowest risk of obesity. The multivariable-adjusted spline regression model further confirmed a stable negative linear relationship between dietary fiber intake and obesity risk (non-linear *p*-value = 0.2016). With increasing dietary fiber intake, the OR curve for obesity exhibits a stable downward trend (Figure 2A). We also conducted an RCS curve analysis stratified by alcohol consumption status and found that the OR curve for obesity in non-drinkers exhibited a more pronounced downward trend with increasing dietary fiber intake compared to drinkers. The above analysis was conducted using the fully adjusted model (Figure 2C). The same conclusion was observed in the non-Hispanic Black population (Figure 2B) and the non-smoking population (Figure 2D). Our study’s findings support current dietary guidelines for increasing fiber intake from whole grains, fruits, vegetables, and legumes, which can be promoted through public health campaigns (36). The results also highlight the potential for clinical dietary interventions, particularly for high-risk individuals, with a focus on achieving at least 20.8 g/day of dietary fiber to reduce obesity risk. Additionally, stratified analyses indicate that the protective effects of fiber against obesity are stronger in non-drinkers and non-smokers, suggesting that alcohol and smoking may diminish fiber’s benefits. These insights emphasize the need to consider lifestyle factors in personalized nutrition recommendations.

**Table 2:** Results from a multiple logistic regression analysis of the association between fiber intake and obesity, weighted.

Variables	Primary model		Model 1		Model 2		Model 3	
	OR (95% CI)	*p*-value	OR (95% CI)	*p*-value	OR (95% CI)	*p*-value	OR (95% CI)	*p*-value
Continuous	0.99(0.98, 0.99)	<0.0001	0.99(0.99, 0.99)	<0.0001	0.99(0.99, 0.99)	<0.0001	0.99(0.98, 0.99)	<0.0001
Fiber intake (quartile)								
Q1	ref		ref		ref		ref	
Q2	0.91(0.85, 0.99)	0.02	0.93(0.86, 1.01)	0.08	0.94(0.86, 1.01)	0.10	0.91(0.84, 0.99)	0.03
Q3	0.82(0.75, 0.89)	<0.0001	0.83(0.77, 0.91)	<0.0001	0.84(0.77, 0.91)	<0.0001	0.82(0.75, 0.89)	<0.0001
Q4	0.73(0.66, 0.80)	<0.0001	0.75(0.69, 0.83)	<0.0001	0.76(0.69, 0.83)	<0.0001	0.74(0.67, 0.83)	<0.0001
*P* for trend		<0.0001		<0.0001		<0.0001		<0.0001

Primary model: adjusted for none. Model 1: The age, sex, and ethnicity of participants were adjusted. Model 2: The age, sex, education level, ethnicity, PIR, and marriage of participants were adjusted. Model 3: The age, sex, education level, ethnicity, PIR, marriage, drinking status, smoking status, hypertension, diabetes mellitus, energy intake, work activity, and recreational activity of participants were adjusted. Q1, ≤9.1 g/day; Q2, 9.1–14.1 g/day; Q3, 14.1–20.8 g/day; Q4, ≥20.8 g/day.

### 3.3 Correlation between dietary fiber intake and outcomes of mortality

**Figure 3:**
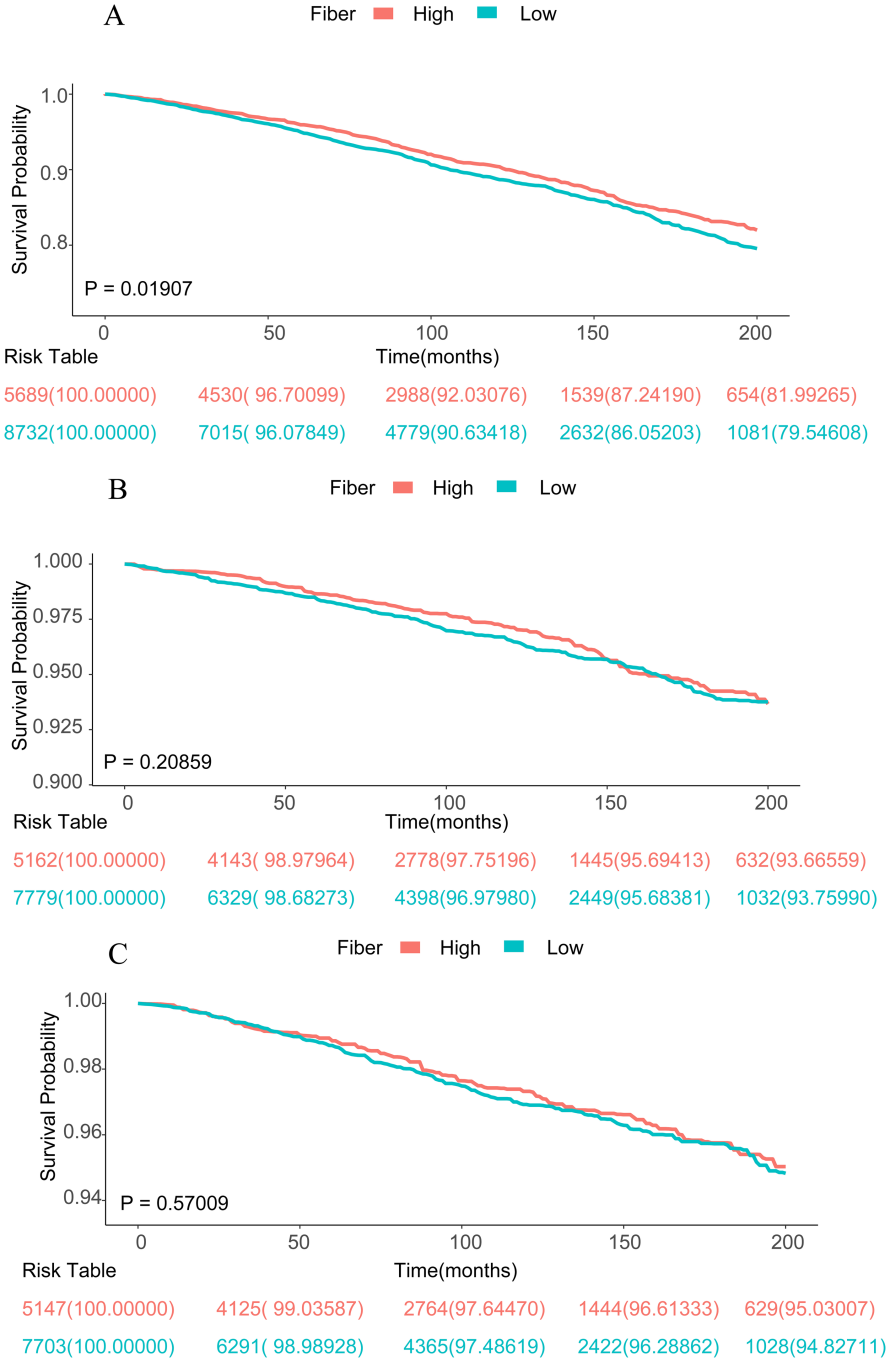
Kaplan–Meier survival curves for mortality outcomes. **(A)** for all-cause mortality, **(B)** for cardiac mortality, and **(C)** for cancer mortality. According to the fiber intake among patients with obesity.

**Figure 4:**
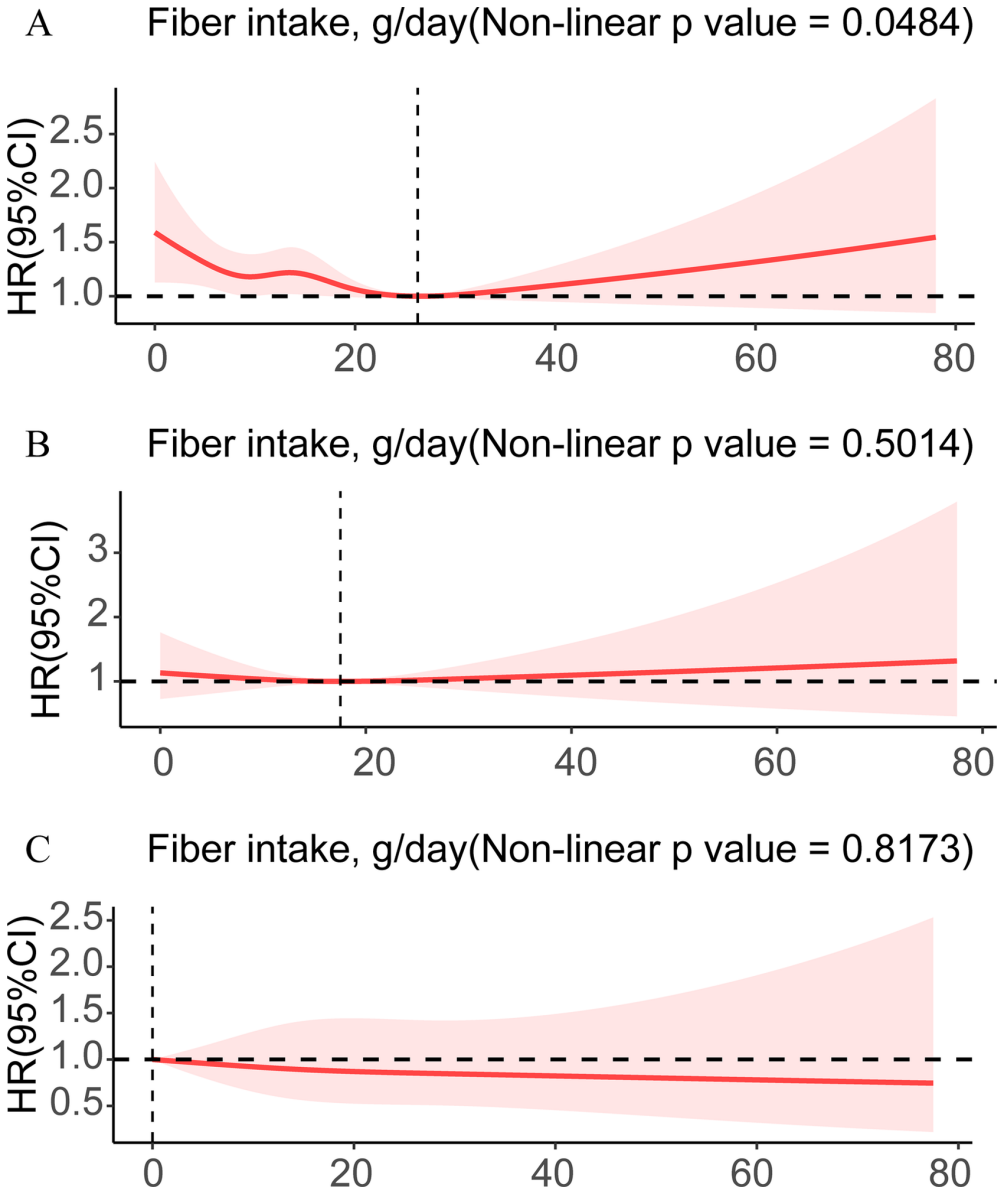
RCS shows the non-linear relationships of fiber with all-cause mortality **(A)**, cardiac mortality **(B)**, and cancer mortality **(C)**.

In the fully adjusted model, results of the multivariable Cox regression model indicated that participants in quartile 4 experienced a 21% reduction in all-cause mortality than those in quartile 1 [hazard ratio (HR) = 0.79; 95% CI, 0.65–0.96, *p* = 0.02]. The same conclusion was observed in the primary model, Model 1, and Model 2 (Table 3). The trend *p*-values for all aforementioned models were less than 0.02, suggesting that with increasing dietary fiber intake, all-cause mortality among obese individuals tended to decrease, thereby enhancing survival rates. Regarding cardiac mortality, we revealed that dietary fiber intake in quartile 4 tremendously reduced it, with a 38% decrease in cardiac mortality than quartile 4 in Model 2 (HR = 0.62; 95% CI, 0.43–0.91, *p* = 0.02). The same conclusion was also demonstrated in the raw model and Model 1. There was no significant correlation between dietary fiber intake and cancer mortality (*p* > 0.05). Furthermore, Kaplan–Meier survival analysis demonstrated that participants were grouped into high and low dietary fiber intake groups. Between the low and high dietary fiber intake groups, Figure 3A shows a statistically significant difference in survival rates for all-cause mortality (*p* = 0.01907), indicating that high dietary fiber intake may improve survival rates among obese individuals. However, Kaplan–Meier survival analyses did not find a statistically significant correlation between dietary fiber intake and either cardiac or cancer mortality (*p* > 0.05; Figures 3B,C). Additionally, the results of the multivariable-adjusted spline regression model illustrated a non-linear U-shaped correlation between all-cause mortality and dietary fiber intake (non-linear *p* < 0.05; Figure 4A), with the HR curve for all-cause mortality initially decreasing and then increasing with increasing dietary fiber intake. Further analysis identified a threshold of 26.3 g/day of dietary fiber intake for the HR curve of all-cause mortality. Nevertheless, no non-linear relationship was found between dietary fiber intake and either cardiac or cancer mortality (non-linear *p* > 0.05; Figures 4B,C). Our study found a 21% lower all-cause mortality in quartile 4 than in quartile 1. Considering obesity’s link to chronic diseases and mortality, higher dietary fiber intake may counteract some obesity-related risks and improve survival. This highlights the importance of promoting sufficient fiber intake for obesity management and public health nutrition.

**Table 3:** Cox regression analysis to identify the association between dietary fiber intake and mortality among obese patients.

Outcome	Primary model		Model 1		Model 2		Model 3	
	HR (95% CI)	*p*-value	HR (95% CI)	*p*-value	HR (95% CI)	*p*-value	HR (95% CI)	*p*-value
All-cause of mortality no. of deaths/patients (2061/14421)								
Fiber intake (quartile)								
Q1	ref		ref		ref		ref	
Q2	0.99(0.83, 1.17)	0.87	0.85(0.72, 1.00)	0.05	0.92(0.77, 1.08)	0.30	0.94(0.80, 1.11)	0.49
Q3	0.94(0.79, 1.13)	0.51	0.80(0.67, 0.95)	0.01	0.86(0.72, 1.03)	0.11	0.90(0.75, 1.08)	0.27
Q4	0.76(0.62, 0.92)	0.005	0.66(0.55, 0.79)	<0.0001	0.74(0.62, 0.89)	0.001	0.79(0.65, 0.96)	0.02
*P* for trend		0.01		<0.0001		0.002		0.02
Cardiac mortality no. of deaths/patients (581/14421)								
Fiber intake (quartile)								
Q1	ref		ref		ref		ref	
Q2	0.93(0.65, 1.34)	0.70	0.78(0.55, 1.12)	0.18	0.85(0.59, 1.23)	0.40	0.93(0.65, 1.33)	0.70
Q3	0.99(0.75, 1.30)	0.92	0.80(0.59, 1.07)	0.14	0.89(0.64, 1.22)	0.45	1.01(0.74, 1.37)	0.97
Q4	0.68(0.47, 0.98)	0.04	0.55(0.37, 0.80)	0.002	0.62(0.43, 0.91)	0.02	0.76(0.50, 1.14)	0.18
*P* for trend		0.06		0.002		0.02		0.3
Cancer mortality no. of deaths/patients (490/14421)								
Fiber intake (quartile)								
Q1	ref		ref		ref		ref	
Q2	0.96(0.72, 1.30)	0.81	0.81(0.61, 1.08)	0.15	0.87(0.65, 1.15)	0.33	0.95(0.70, 1.29)	0.74
Q3	0.98(0.70, 1.38)	0.92	0.83(0.60, 1.15)	0.26	0.90(0.66, 1.24)	0.53	1.02(0.74, 1.41)	0.89
Q4	0.82(0.59, 1.15)	0.24	0.66(0.47, 0.93)	0.02	0.74(0.52, 1.03)	0.08	0.89(0.61, 1.29)	0.54
*P* for trend		0.33		0.03		0.13		0.71

Primary model: adjusted for none. Model 1: The age, sex, and ethnicity of participants were adjusted. Model 2: The age, sex, education level, ethnicity, PIR, and marriage of participants were adjusted. Model 3: The age, sex, education level, ethnicity, PIR, marriage, drinking status, smoking status, hypertension, diabetes mellitus, energy intake, work activity, and recreational activity of participants were adjusted. Q1, ≤9.1 g/day; Q2, 9.1–14.1 g/day; Q3, 14.1–20.8 g/day; Q4, ≥20.8 g/day.

### 3.4 Subgroup analysis

**Figure 5:**
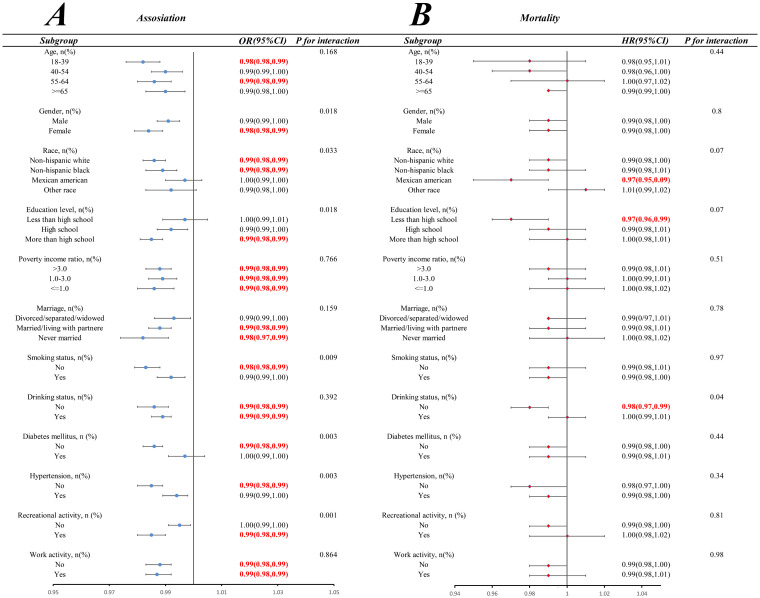
Subgroup analyses of the association of fiber intake with obesity **(A)** and all-cause mortality **(B)** among patients with obesity.

We conducted stratified weighted multiple regression analysis for subgroup analysis by age, hypertension, gender, education level, work activities, marital status, alcohol consumption, recreational activities, smoking status, ethnicity, diabetes, and PIR, to further investigate the correlation between obesity across diverse populations and fiber intake. The subgroup analysis depicted in Figure 5A presented the correlation between fiber intake and obesity incidence, and analyzed the interaction between categorical variables and dietary fiber intake. A consistent negative correlation between fiber intake and obesity incidence across different demographic characteristics, lifestyles, and disease statuses was shown in the results (with most interaction *p*-values>0.01). However, we found significant interactions between fiber intake and smoking status, diabetes status, hypertension status, and recreational activities (with interaction *p*-values<0.01). Figure 5B depicts the subgroup analysis of the correlation between all-cause mortality and dietary fiber intake, and shows a consistent negative correlation between fiber intake and all-cause mortality across different demographic characteristics, lifestyles, and disease statuses (with all interaction *p*-values>0.05).

## 4 Discussion

Dietary fiber is a significant factor in maintaining health. It was primarily found in foods such as legumes, vegetables, fruits, and grains (37). Dietary fiber is not only famous for preventing constipation (38) but also produces other beneficial effects. Although many studies have focused on the potential effects associated with dietary fiber intake, considering both the effects on the body when deficient and when supplemented with adequate amounts, there remains a gap in research on the effect of dietary fiber intake on obesity incidence. In this cross-sectional study, which included 39,184 participants, we illustrated that increased dietary fiber intake was linked with a lower incidence of obesity, as obesity can lead to many associated diseases that result in patient death. Thus, we further explored the correlation between different causes of mortality in obese patients and dietary fiber intake, finding that in obese patients, low all-cause mortality was significantly linked with high dietary fiber intake, which is vital for improving the prognosis of obese patients.

Our study results demonstrate a stable inverse association between dietary fiber intake and obesity prevalence, with maximal preventive effect observed at intake levels exceeding 20.8 g/day. These findings align with previous research. The cross-sectional study by Efrem et al. indicates that high dietary fiber intake is associated with reduced obesity risk, particularly among male participants and older adults (39). Focusing on Japanese patients with type 2 diabetes and stratified by sex and age, their study revealed sex- and age-specific associations between dietary fiber intake and obesity, providing a precise direction for interventions in specific populations. In contrast, our study included 39,184 US adults. The large, nationally representative sample enhanced statistical power. Our study employed a more comprehensive analytical approach, including multivariable logistic regression to analyze obesity incidence, Cox regression for all-cause mortality, RCS to quantify dose–response relationships, and Kaplan–Meier survival analysis and subgroup analyses to strengthen the robustness of the results. Similarly, Derek C Miketinas et al. clearly confirmed through a rigorous randomized controlled design and multivariate analysis that in calorie-restricted diets, increased dietary fiber intake is significantly negatively correlated with weight loss (40). However, the study only included 345 overweight or obese adults, with a relatively small sample size. Moreover, the study focused on weight loss effects and dietary adherence, without involving mortality or other long-term health indicators. By contrast, our study analyzed both obesity incidence and all-cause mortality, providing in-depth insights into obesity prevention and survival prognosis, thus bearing broader public health significance. Additionally, our stratified RCS analysis results indicated that, compared to smokers, the OR curve for obesity in non-smokers presented a more significant downward trend as dietary fiber intake increased, and the same trend was observed in non-Hispanic Black populations and non-drinkers. Our study suggests that in obese populations, racial background and lifestyle factors can influence the preventive effects of dietary fiber against obesity, with greater benefits observed among those who neither smoke nor drink alcohol. In addition, the Cox regression analysis results found that high dietary fiber intake can reduce the all-cause mortality risk in obese individuals and improve their survival rate. RCS analyses showed a non-linear U-shaped correlation between all-cause mortality and dietary fiber intake (*p* < 0.05), with the HR for all-cause mortality decreasing and then increasing. Further analysis of the HR curve for all-cause mortality identified a threshold of 26.3 g/day of dietary fiber intake. However, no significant association was found between dietary fiber intake and mortality from heart disease or cancer (*p* > 0.05). Our findings provide quantitative guidance for dietary fiber intake in obese populations, indicating that all-cause mortality risk is minimized when intake approaches the threshold of 26.3 g/day. Public health implementation should integrate this evidence with tailored recommendations for individualized management.

The adverse correlation between the incidence of obesity and increased dietary fiber intake may be achieved through several mechanisms. First, dietary fiber increases food bulk and satiety. It delays gastric emptying, thereby reducing the amount of food eaten at the next meal (41). It regulates the secretion of intestinal hormones and enhances satiety (42). In addition, short-chain fatty acids, enhanced with the addition of dietary fiber intake, ultimately suppress appetite (43). It also increases insulin sensitivity and energy expenditure. Consistently, a recent 16-week intervention study of overweight and obese children using oligofructose-rich inulin reduced their fat mass (44). Dietary fiber intake significantly reduced levels of inflammatory markers in the body (45). Dietary fiber can manipulate the gastrointestinal enteroendocrine pathway to improve metabolic function (46). Dietary fiber modulates intestinal alkaline phosphatase enzyme activity (47). Dietary fiber can both lower serum cholesterol and reduce cholesterol absorption (48). The above studies have focused on the mechanism of dietary fiber in humans, but they have not verified whether dietary fiber intake is effective in reducing the incidence of obesity. Therefore, our study provides new evidence regarding the impact of dietary fiber intake on the incidence of obesity.

Our study offers valuable insights into the relationship between dietary fiber intake and obesity. However, it also has some inevitable limitations. First, as a cross-sectional study, our research does not establish definitive causal relationships between dietary fiber intake and obesity incidence. Furthermore, the correlations identified in the study do not necessarily possess universal applicability. Second, although we adjusted for numerous known confounding factors through multivariable models, it is impossible to completely rule out the possibility that unknown or unmeasured confounding factors may influence the observed associations. Third, dietary fiber intake was assessed using the 24-h dietary recall method, which is prone to recall bias and measurement errors. Although NHANES employs standardized approaches and calculates average intakes to minimize errors, the inherent imprecision of self-reported data remains a significant limitation. Despite the aforementioned limitations, our study demonstrates that dietary fiber intake plays a positive role in preventing obesity and improving survival rates among obese individuals. The above findings provide a theoretical basis for the precise dietary management of obese patients and are of guiding value for the formulation of clinical nutrition intervention strategies. To confirm these findings and explore their potential in obesity prevention and management, future longitudinal studies and randomized controlled trials are warranted.

## 5 Conclusion

In summary, we illustrated that increased dietary fiber intake was significantly linked with a decreased probability of obesity incidence. In particular, non-drinkers showed a more pronounced downward trend in obesity events compared with drinkers, and the same findings were observed in non-Hispanic Black populations and non-smoking populations. Moreover, we exhibited that high dietary fiber intake may be linked with lower mortality in obese patients. Thus, diets rich in dietary fiber may lead to a reduction in the incidence of obesity, as well as in all-cause mortality in obese patients.

## Data Availability

The datasets presented in this study can be found in online repositories. The names of the repository/repositories and accession number(s) can be found below: data is provided within the manuscript or supplementary information files. NHANES data can be downloaded for free from the following website: https://www.cdc.gov/nchs/nhanes/.

## Footnotes

^1^ https://wwwn.cdc.gov/nchs/nhanes/continuousnhanes/questionnaires.aspx?Cycle=2019-2020
