# Supplementary material for: Association between dietary fiber intake and obesity in US adults: from NHANES 1999–2018
Source: Front Nutr. 2025 Jul 7;12:1602600. doi: 10.3389/fnut.2025.1602600 (PMC12277132; doi:10.3389/fnut.2025.1602600)
Supplement: Supplementary file 1 [file Table_1.docx]

Table S1 Clinical characteristics of obesity patients.

| Characteristic | Overall | Alive | Dead | P-value |
| --- | --- | --- | --- | --- |
| N | 14421 | 12359 | 2062 |  |
| Age, years | 48.15(0.23) | 46.21(0.23) | 63.61(0.45) | < 0.0001 |
| Body Mass Index(kg/m^2^) | 28.80(0.07) | 24.86(0.03) | 35.86(0.08) | < 0.0001 |
| Sex, n (%) |  |  |  | < 0.001 |
| Female | 7889(52.69) | 6898(53.24) | 991(48.26) |  |
| Male | 6532(47.31) | 5461(46.76) | 1071(51.74) |  |
| Race, n (%) |  |  |  | < 0.0001 |
| Non-hispanic white | 6302(68.04) | 5116(66.85) | 1186(77.58) |  |
| Non-hispanic black | 3657(13.56) | 3188(13.72) | 469(12.26) |  |
| Mexican american | 2638(8.90) | 2369(9.51) | 269(4.09) |  |
| Other race | 1824(9.49) | 1686(9.92) | 138(6.07) |  |
| Education level, n (%) |  |  |  | < 0.0001 |
| Less than high school | 1538(5.00) | 1139(4.09) | 399(12.25) |  |
| High school | 4255(26.28) | 3408(24.56) | 847(39.94) |  |
| More than high school | 8628(68.72) | 7812(71.34) | 816(47.82) |  |
| Poverty income ratio, n (%) |  |  |  | < 0.0001 |
| <=1.0 | 2970(14.66) | 2545(14.34) | 425(17.19) |  |
| 1.0–3.0 | 5150(47.51) | 4619(49.27) | 531(33.45) |  |
| >3.0 | 6301(37.83) | 5195(36.39) | 1106(49.36) |  |
| Marriage, n (%) |  |  |  | < 0.0001 |
| Divorced/separated/widowed | 3436(20.29) | 2639(18.34) | 797(35.83) |  |
| Married/living with partner | 8696(63.29) | 7582(64.24) | 1114(55.75) |  |
| Never married | 2289(16.42) | 2138(17.42) | 151( 8.42) |  |
| Drinking status, n (%) |  |  |  | < 0.0001 |
| No | 5004(28.88) | 3884(26.37) | 1120(48.94) |  |
| Yes | 9417(71.12) | 8475(73.63) | 942(51.06) |  |
| Smoking status, n (%) |  |  |  | < 0.0001 |
| No | 7836(53.57) | 7000(55.61) | 836(37.34) |  |
| Yes | 6585(46.43) | 5359(44.39) | 1226(62.66) |  |
| Diabetes mellitus, n (%) |  |  |  | < 0.0001 |
| No | 10618(78.28) | 9474(80.70) | 1144(58.98) |  |
| Yes | 3803(21.72) | 2885(19.30) | 918(41.02) |  |
| Hypertension, n (%) |  |  |  | < 0.0001 |
| No | 6591(49.45) | 6152(52.67) | 439(23.71) |  |
| Yes | 7830(50.55) | 6207(47.33) | 1623(76.29) |  |
| Work activity, n (%) |  |  |  | < 0.0001 |
| No | 7420(46.08) | 6131(44.76) | 1289(56.64) |  |
| Yes | 7001(53.92) | 6228(55.24) | 773(43.36) |  |
| Recreational activity, n (%) |  |  |  | < 0.0001 |
| No | 9336(61.27) | 7652(59.03) | 1684(79.09) |  |
| Yes | 5085(38.73) | 4707(40.97) | 378(20.91) |  |
| Energy intake (kcals/day) | 2145.38(11.80) | 2178.58(12.27) | 1880.66(33.23) | < 0.0001 |
| Fiber intake (g/day) | 15.80(0.14) | 15.92(0.15) | 14.83(0.34) | 0.004 |

Continuous variables are shown as weighted means ± standard errors.

Categorical variables are shown as unweighted counts (weighted percentages).
